# Bis(acridine-κ*N*)dibromidoplatinum(II)

**DOI:** 10.1107/S160053681003309X

**Published:** 2010-08-21

**Authors:** Kwang Ha

**Affiliations:** a School of Applied Chemical Engineering, The Research Institute of Catalysis, Chonnam National University, Gwangju 500-757, Republic of Korea

## Abstract

In the title complex, [PtBr_2_(C_13_H_9_N)_2_], the Pt^II^ ion is four-coordinated in a slightly distorted square-planar environment by two N atoms from two acridine ligands and two Br atoms. The Pt atom is located on an inversion centre, and thus the asymmetric unit contains one half of the complex and the PtN_2_Br_2_ unit is exactly planar. The dihedral angle between the PtN_2_Br_2_ unit and acridine ligand is 78.98 (9)°. In the crystal structure, the complex mol­ecules are arranged in two distinct chains along [110] and [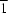
10]. In the chains, inter­molecular π–π inter­actions between the pyridyl and benzene rings connect the complex mol­ecules, with a centroid–centroid distance of 3.631 (4) Å.

## Related literature

For the crystal structure of [PtCl_2_(acridine)_2_], see: Ha (2010 ▶). For the formation of polymorphs of acridine using dicarb­oxy­lic acids, see: Mei & Wolf (2004 ▶).
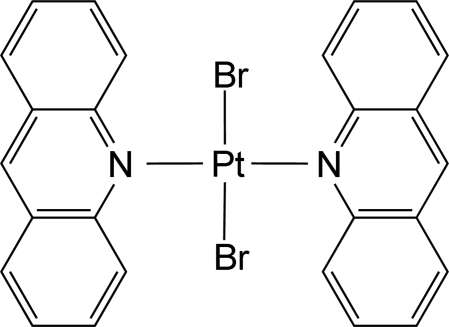


## Experimental

### 

#### Crystal data

[PtBr_2_(C_13_H_9_N)_2_]
*M* *_r_* = 713.33
Monoclinic, 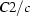

*a* = 16.0256 (9) Å
*b* = 8.6845 (5) Å
*c* = 17.0646 (10) Å
β = 115.017 (1)°
*V* = 2152.1 (2) Å^3^
*Z* = 4
Mo *K*α radiation
μ = 10.25 mm^−1^
*T* = 200 K
0.35 × 0.06 × 0.04 mm

#### Data collection

Bruker SMART 1000 CCD diffractometer
Absorption correction: multi-scan (*SADABS*; Bruker, 2001 ▶) *T*_min_ = 0.601, *T*_max_ = 1.000
6467 measured reflections
2091 independent reflections
1672 reflections with *I* > 2σ(*I*)
*R*_int_ = 0.048

#### Refinement

*R*[*F* ^2^ > 2σ(*F* ^2^)] = 0.031
*wR*(*F* ^2^) = 0.070
*S* = 1.00
2091 reflections
142 parameters
H-atom parameters constrained
Δρ_max_ = 2.19 e Å^−3^
Δρ_min_ = −0.92 e Å^−3^

### 

Data collection: *SMART* (Bruker, 2007 ▶); cell refinement: *SAINT* (Bruker, 2007 ▶); data reduction: *SAINT*; program(s) used to solve structure: *SHELXS97* (Sheldrick, 2008 ▶); program(s) used to refine structure: *SHELXL97* (Sheldrick, 2008 ▶); molecular graphics: *ORTEP-3* (Farrugia, 1997 ▶) and *PLATON* (Spek, 2009 ▶); software used to prepare material for publication: *SHELXL97*.

## Supplementary Material

Crystal structure: contains datablocks global, I. DOI: 10.1107/S160053681003309X/hy2340sup1.cif

Structure factors: contains datablocks I. DOI: 10.1107/S160053681003309X/hy2340Isup2.hkl

Additional supplementary materials:  crystallographic information; 3D view; checkCIF report

## Figures and Tables

**Table 1:** Selected bond lengths (Å)

Pt1—N1	2.058 (4)
Pt1—Br1	2.4385 (7)

**Fig. 1.**
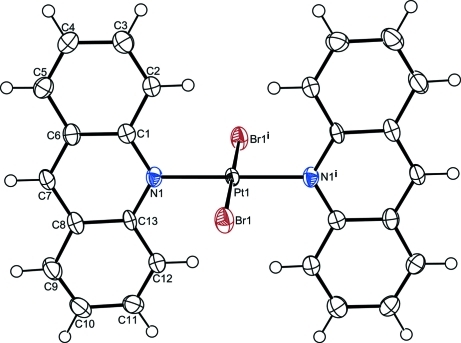
The molecular structure of the title complex. Displacement ellipsoids are drawn at the 40% probability level. [Symmetry code: (i) 1-x, 1-y, 1-z.]

**Fig. 2.**
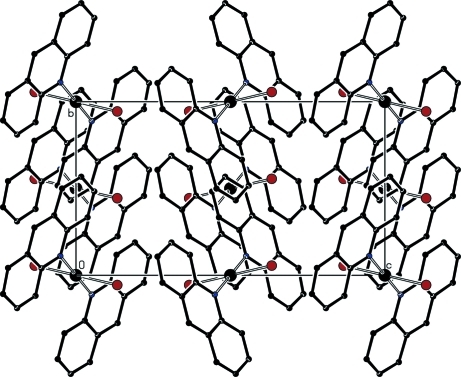
View of the unit-cell contents of the title complex. H atoms have been omitted for clarity.

## supplementary crystallographic information

### Comment

The title complex, [PtBr_2_(acr)_2_] (acr = acridine), is isomorphous with the chlorido analogue [PtCl_2_(acr)_2_] (Ha, 2010). In the complex, the Pt^II^ ion is four-coordinated in an essentially square-planar environment by two N atoms from two acridine ligands and two Br atoms (Table 1 and Fig. 1). The Pt atom is located on an inversion centre, and thus the asymmetric unit contains one half of the complex and the PtN_2_Br_2_ unit is exactly planar. The nearly planar acridine ligands, with a maximum deviation of 0.072 (6) Å (C11) from the least-squares plane, are parallel. The dihedral angle between the PtN_2_Br_2_ unit and acridine ligand is 78.98 (9)°. The Br atoms are in a *trans* arrangement and almost perpendicular to the acridine planes, with the bond angle N1—Pt1—Br1 = 88.65 (14)°. In the crystal structure, the complex molecules are arranged in two distinct chains along [1 1 0] and [1 1 0] (Fig. 2). In the chains, intermolecular π–π interactions between the pyridyl and benzene rings connect the complex molecules, with a centroid–centroid distance of 3.631 (4) Å, and the dihedral angle between the ring planes is 1.2 (3)°. The packing pattern is considerably similar to that of the most stable polymorph of acridine (Mei & Wolf, 2004).

### Experimental

To a solution of K_2_PtBr_4_ (0.203 g, 0.342 mmol) in H_2_O (30 ml) was added acridine (0.131 g, 0.730 mmol) and the mixture was refluxed for 3 h. The precipitate was then separated by filtration, washed with H_2_O and EtOH and dried under vacuum to give a yellow powder (0.186 g). Crystals suitable for X-ray analysis were obtained by slow evaporation from an *N,N*-dimethylformamide solution at 323 K.

### Refinement

H atoms were positioned geometrically and allowed to ride on their respective parent atoms [C—H = 0.95 Å and *U*_iso_(H) = 1.2*U*_eq_(C)]. The highest peak (2.19 e Å^-3^) and the deepest hole (-0.92 e Å^-3^) in the difference Fourier map are located 1.04 and 0.76 Å from the Pt1 atom, respectively.

### Crystal data

**Table:**

[PtBr_2_(C_13_H_9_N)_2_]	*F*(000) = 1344
*M**_r_* = 713.33	*D*_x_ = 2.202 Mg m^−^^3^
Monoclinic, *C*2/*c*	Mo *K*α radiation, λ = 0.71073 Å
Hall symbol: -C 2yc	Cell parameters from 3125 reflections
*a* = 16.0256 (9) Å	θ = 2.7–26.0°
*b* = 8.6845 (5) Å	µ = 10.25 mm^−^^1^
*c* = 17.0646 (10) Å	*T* = 200 K
β = 115.017 (1)°	Rod, yellow
*V* = 2152.1 (2) Å^3^	0.35 × 0.06 × 0.04 mm
*Z* = 4	

### Data collection

**Table:**

Bruker SMART 1000 CCD diffractometer	2091 independent reflections
Radiation source: fine-focus sealed tube	1672 reflections with *I* > 2σ(*I*)
graphite	*R*_int_ = 0.048
φ and ω scans	θ_max_ = 26.0°, θ_min_ = 2.6°
Absorption correction: multi-scan (*SADABS*; Bruker, 2001)	*h* = −19→19
*T*_min_ = 0.601, *T*_max_ = 1.000	*k* = −10→10
6467 measured reflections	*l* = −14→21

### Refinement

**Table:**

Refinement on *F*^2^	Primary atom site location: structure-invariant direct methods
Least-squares matrix: full	Secondary atom site location: difference Fourier map
*R*[*F*^2^ > 2σ(*F*^2^)] = 0.031	Hydrogen site location: inferred from neighbouring sites
*wR*(*F*^2^) = 0.070	H-atom parameters constrained
*S* = 1.00	*w* = 1/[σ^2^(*F*_o_^2^) + (0.0286*P*)^2^] where *P* = (*F*_o_^2^ + 2*F*_c_^2^)/3
2091 reflections	(Δ/σ)_max_ < 0.001
142 parameters	Δρ_max_ = 2.19 e Å^−^^3^
0 restraints	Δρ_min_ = −0.92 e Å^−^^3^

### Fractional atomic coordinates and isotropic or equivalent isotropic displacement parameters (Å^2^)

**Table:**

	*x*	*y*	*z*	*U*_iso_*/*U*_eq_	
Pt1	0.5000	0.5000	0.5000	0.02062 (12)	
Br1	0.51478 (4)	0.44407 (8)	0.36640 (5)	0.03216 (18)	
N1	0.3748 (3)	0.3887 (5)	0.4493 (3)	0.0218 (11)	
C1	0.3686 (4)	0.2391 (6)	0.4712 (4)	0.0215 (13)	
C2	0.4437 (4)	0.1654 (6)	0.5371 (4)	0.0267 (15)	
H2	0.4997	0.2201	0.5666	0.032*	
C3	0.4373 (4)	0.0170 (6)	0.5592 (5)	0.0326 (16)	
H3	0.4891	−0.0305	0.6036	0.039*	
C4	0.3550 (4)	−0.0683 (7)	0.5172 (4)	0.0299 (15)	
H4	0.3519	−0.1725	0.5328	0.036*	
C5	0.2809 (4)	−0.0004 (6)	0.4549 (5)	0.0300 (15)	
H5	0.2252	−0.0569	0.4275	0.036*	
C6	0.2850 (4)	0.1547 (7)	0.4295 (4)	0.0256 (14)	
C7	0.2099 (4)	0.2251 (6)	0.3653 (4)	0.0243 (14)	
H7	0.1546	0.1688	0.3358	0.029*	
C8	0.2149 (4)	0.3780 (7)	0.3437 (4)	0.0232 (14)	
C9	0.1382 (4)	0.4572 (7)	0.2798 (5)	0.0339 (17)	
H9	0.0824	0.4031	0.2483	0.041*	
C10	0.1441 (4)	0.6080 (7)	0.2638 (4)	0.0334 (16)	
H10	0.0921	0.6598	0.2220	0.040*	
C11	0.2273 (4)	0.6896 (7)	0.3088 (5)	0.0347 (16)	
H11	0.2305	0.7956	0.2968	0.042*	
C12	0.3023 (4)	0.6182 (7)	0.3687 (4)	0.0283 (15)	
H12	0.3574	0.6750	0.3984	0.034*	
C13	0.2995 (4)	0.4596 (6)	0.3878 (4)	0.0232 (14)	

### Atomic displacement parameters (Å^2^)

**Table:**

	*U*^11^	*U*^22^	*U*^33^	*U*^12^	*U*^13^	*U*^23^
Pt1	0.01485 (17)	0.02395 (18)	0.0197 (2)	−0.00426 (13)	0.00406 (14)	−0.00171 (15)
Br1	0.0278 (3)	0.0431 (4)	0.0263 (4)	−0.0104 (3)	0.0121 (3)	−0.0085 (3)
N1	0.017 (2)	0.024 (3)	0.023 (3)	−0.0023 (19)	0.007 (2)	−0.004 (2)
C1	0.019 (3)	0.026 (3)	0.020 (4)	0.001 (2)	0.009 (3)	−0.002 (3)
C2	0.022 (3)	0.030 (3)	0.024 (4)	−0.005 (3)	0.005 (3)	−0.002 (3)
C3	0.029 (3)	0.033 (4)	0.033 (4)	0.004 (3)	0.011 (3)	0.002 (3)
C4	0.034 (4)	0.024 (3)	0.034 (4)	−0.003 (3)	0.016 (3)	0.000 (3)
C5	0.031 (3)	0.030 (3)	0.035 (4)	−0.005 (3)	0.020 (3)	−0.003 (3)
C6	0.021 (3)	0.030 (3)	0.028 (4)	−0.001 (3)	0.012 (3)	−0.004 (3)
C7	0.015 (3)	0.032 (3)	0.024 (4)	−0.005 (2)	0.005 (3)	−0.004 (3)
C8	0.017 (3)	0.031 (3)	0.020 (4)	0.002 (2)	0.006 (3)	−0.004 (3)
C9	0.022 (3)	0.039 (4)	0.032 (4)	0.002 (3)	0.003 (3)	−0.002 (3)
C10	0.028 (4)	0.044 (4)	0.022 (4)	0.004 (3)	0.004 (3)	0.005 (3)
C11	0.039 (4)	0.033 (4)	0.028 (4)	0.000 (3)	0.010 (3)	0.003 (3)
C12	0.019 (3)	0.033 (3)	0.027 (4)	−0.003 (3)	0.003 (3)	0.004 (3)
C13	0.016 (3)	0.030 (4)	0.020 (4)	−0.005 (2)	0.005 (3)	−0.006 (3)

### Geometric parameters (Å, °)

**Table:**

Pt1—N1	2.058 (4)	C6—C7	1.380 (8)
Pt1—Br1	2.4385 (7)	C7—C8	1.389 (8)
N1—C13	1.366 (7)	C7—H7	0.9500
N1—C1	1.367 (7)	C8—C9	1.428 (8)
C1—C2	1.406 (8)	C8—C13	1.430 (7)
C1—C6	1.426 (7)	C9—C10	1.349 (9)
C2—C3	1.359 (8)	C9—H9	0.9500
C2—H2	0.9500	C10—C11	1.415 (8)
C3—C4	1.415 (9)	C10—H10	0.9500
C3—H3	0.9500	C11—C12	1.355 (8)
C4—C5	1.349 (9)	C11—H11	0.9500
C4—H4	0.9500	C12—C13	1.420 (8)
C5—C6	1.425 (8)	C12—H12	0.9500
C5—H5	0.9500		
			
N1—Pt1—N1^i^	180.00 (16)	C7—C6—C5	121.6 (5)
N1—Pt1—Br1^i^	91.35 (14)	C7—C6—C1	119.2 (5)
N1^i^—Pt1—Br1^i^	88.65 (14)	C5—C6—C1	119.2 (5)
N1—Pt1—Br1	88.65 (14)	C6—C7—C8	120.3 (5)
N1^i^—Pt1—Br1	91.35 (14)	C6—C7—H7	119.8
Br1^i^—Pt1—Br1	180.0	C8—C7—H7	119.8
C13—N1—C1	119.7 (5)	C7—C8—C9	122.4 (5)
C13—N1—Pt1	119.9 (4)	C7—C8—C13	118.8 (5)
C1—N1—Pt1	120.1 (4)	C9—C8—C13	118.8 (5)
N1—C1—C2	120.9 (5)	C10—C9—C8	120.7 (6)
N1—C1—C6	120.9 (5)	C10—C9—H9	119.7
C2—C1—C6	118.1 (5)	C8—C9—H9	119.7
C3—C2—C1	120.9 (6)	C9—C10—C11	120.4 (6)
C3—C2—H2	119.5	C9—C10—H10	119.8
C1—C2—H2	119.5	C11—C10—H10	119.8
C2—C3—C4	121.3 (6)	C12—C11—C10	120.8 (6)
C2—C3—H3	119.3	C12—C11—H11	119.6
C4—C3—H3	119.3	C10—C11—H11	119.6
C5—C4—C3	119.4 (6)	C11—C12—C13	120.9 (5)
C5—C4—H4	120.3	C11—C12—H12	119.6
C3—C4—H4	120.3	C13—C12—H12	119.6
C4—C5—C6	121.0 (6)	N1—C13—C12	120.7 (5)
C4—C5—H5	119.5	N1—C13—C8	120.9 (5)
C6—C5—H5	119.5	C12—C13—C8	118.3 (5)
			
Br1^i^—Pt1—N1—C13	104.3 (4)	C5—C6—C7—C8	178.0 (6)
Br1—Pt1—N1—C13	−75.7 (4)	C1—C6—C7—C8	−2.1 (9)
Br1^i^—Pt1—N1—C1	−81.7 (4)	C6—C7—C8—C9	−177.9 (6)
Br1—Pt1—N1—C1	98.3 (4)	C6—C7—C8—C13	1.2 (9)
C13—N1—C1—C2	−177.0 (6)	C7—C8—C9—C10	176.4 (6)
Pt1—N1—C1—C2	8.9 (8)	C13—C8—C9—C10	−2.7 (10)
C13—N1—C1—C6	1.2 (8)	C8—C9—C10—C11	1.4 (10)
Pt1—N1—C1—C6	−172.8 (4)	C9—C10—C11—C12	−0.1 (11)
N1—C1—C2—C3	179.6 (6)	C10—C11—C12—C13	0.2 (10)
C6—C1—C2—C3	1.3 (9)	C1—N1—C13—C12	175.2 (6)
C1—C2—C3—C4	−0.4 (10)	Pt1—N1—C13—C12	−10.8 (8)
C2—C3—C4—C5	−1.0 (10)	C1—N1—C13—C8	−2.1 (9)
C3—C4—C5—C6	1.3 (10)	Pt1—N1—C13—C8	171.9 (4)
C4—C5—C6—C7	179.5 (6)	C11—C12—C13—N1	−178.9 (6)
C4—C5—C6—C1	−0.4 (10)	C11—C12—C13—C8	−1.5 (9)
N1—C1—C6—C7	0.9 (9)	C7—C8—C13—N1	1.0 (9)
C2—C1—C6—C7	179.2 (6)	C9—C8—C13—N1	−179.9 (6)
N1—C1—C6—C5	−179.2 (6)	C7—C8—C13—C12	−176.4 (6)
C2—C1—C6—C5	−0.9 (9)	C9—C8—C13—C12	2.7 (9)

Symmetry codes: (i) −*x*+1, −*y*+1, −*z*+1.
